# Using Mobile Health Technology to Improve HIV Care for Persons Living with HIV and Substance Abuse

**DOI:** 10.1155/2013/194613

**Published:** 2013-12-05

**Authors:** Gregory D. Kirk, Seth S. Himelhoch, Ryan P. Westergaard, Curt G. Beckwith

**Affiliations:** ^1^Departments of Epidemiology & Medicine, Johns Hopkins University, Baltimore, MD 21205, USA; ^2^Department of Psychiatry, University of Maryland, Baltimore, MD 21201, USA; ^3^Division of Infectious Diseases, University of Wisconsin-Madison, Madison, WI 53792, USA; ^4^Division of Infectious Diseases, The Miriam Hospital/Alpert Medical School of Brown University, Providence, RI 02912, USA

Mobile phone technology has a growing and pervasive influence on society, with >6 billion mobile phone subscribers worldwide. Round-the-clock connectedness increasingly represents the norm and smartphones are ubiquitous in many sectors of society. As costs for these technologies decrease, expansion into populations with more limited resources is increasing. With many persons now routinely carrying a portable computing and communication device, adaptation of these mobile technologies for monitoring or improving health has been touted as a revolution in health care [1, 2]. Mobile health, or mHealth, has been broadly defined as medical or public health practice supported by mobile devices. The purpose of this special issue is to provide a research and implementation update on the incorporation of mHealth approaches to improve health outcomes among persons living with HIV/AIDS (PLWHA) and substance use.

PLWHA who are drug and alcohol users face many health challenges including delayed HIV diagnosis, delayed linkage to care, difficulty with adherence to antiretroviral treatment (ART), poorer virologic control, and difficulties with long-term retention in care, compared to nonsubstance users [3, 4]. Substance use may often be associated with other barriers to optimal care (e.g., mental illness, financial and legal difficulties, and inadequate housing and transportation) [5]. Several strategies to improve HIV care among persons with substance abuse have been identified (e.g., multidisciplinary case management, peer navigation) but often require substantial resources. Novel, affordable, and evidence-based strategies that are feasible to implement are needed to identify substance-using persons at risk for poor outcomes, promote engagement in HIV care, enhance adherence, and improve treatment responses.

As with most chronic diseases that rely on adherence to prescribed medical regimens or lifestyle modifications, the underpinnings of optimal HIV care include enhanced capacity for self-management by patients. mHealth strategies hold great promise to transform the approach to HIV care. Real-time monitoring of PLWHA with mHealth devices can generate dynamic, individualized models of behavior and provide a foundation to inform health behavior change strategies. Technology can empower patients, with direct delivery of individualized motivation, education, and support. mHealth strategies will likely be significantly represented in evolving models of care delivery. Wireless technologies remove the barriers of time and distance between patients and providers; this is especially important for substance-using PLWHA and other populations that are hard to reach and difficult to keep engaged in care. Hopefully, mHealth approaches will facilitate HIV care providers to work more effectively and be able to collect higher quality data related to care delivery and health outcomes. 

While mHealth holds great promise, this nascent field remains in early stages of development [6]. Current mHealth interventions to support HIV care have been largely centered in resource-limited countries. Strategies most widely employed have focused on supporting care providers in remote settings and providing text message support to HIV patients. A growing evidence base indicates that weekly text messages can improve ART adherence and viral suppression [7]. Text messaging appears to work best when the message is individually tailored, context-sensitive, and associated with follow-up intervention. In response to these accumulating data, UNAIDS has recommended that mHealth approaches be incorporated into national HIV care programs. As the next step, smartphone interventions will expand the ability to collect intensive, real-time data from patients, provide tailored education or counseling, and facilitate responsive, interactive communication. 

To date, mHealth studies which focus on HIV-infected populations with substance abuse have been limited. In this special issue, eight manuscripts provide an assessment of the scope of mHealth strategies employed among marginalized, substance-using PLWHA. Consistent with the current state of the field, the studies largely represent early stage evaluations of applying novel technology in diverse populations. 

Conducting research studies among vulnerable populations such as persons with HIV or substance abuse raises several ethical issues. Further, mHealth approaches that collect data from and/or deliver interventions to participants during their daily routine also pose unique ethical considerations. Dr. A. B. Labrique and colleagues apply a widely accepted ethical framework to describe a range of ethical issues salient to protection of participants in mHealth research on HIV/AIDS and substance abuse.

Several manuscripts in this special issue report on the acceptability, feasibility, and implementation of mHealth approaches. Dr. C. W. T. Miller and Dr. S. Himelhoch report high levels of ownership and interest in using mobile phones among patients at an urban HIV clinic to help support and improve adherence to ART. Dr. K. J. Horvath and colleagues report that while methamphetamine-using men who have sex with men (MSM) had poorer engagement in HIV care, their use of social media and mobile phone technologies was comparable to nonstimulant using MSM, thus raising the possibility of using mHealth in this population. 

A major focus of mHealth interventions among substance-using PLWHA is to facilitate ART adherence. Among methamphetamine users studied by Dr. D. J. Moore and colleagues, daily texts were sent to assess drug use and ART adherence. In the preliminary analysis, the proportion of texts responded to and both qualitative and quantitative assessments of acceptability indicate favorable responses by participants. Among HIV-infected IDUs receiving ART in China, Dr. M. B. DeSilva and colleagues evaluated real-time, wireless adherence monitoring using the Wisepill system. Overall, their findings were promising with only minor technical issues and participant feedback highlighting the need for unobtrusive technologies. 

Novel intervention strategies which can capture and respond to illicit drug use in realtime can provide a framework for improving engagement and adherence to HIV care. Dr. K. A. Phillips and colleagues report preliminary data from a study of video-based, risk reduction messaging delivered through a smartphone to opioid-dependent patients in drug treatment. Nested within a larger mHealth study in this population, the authors demonstrated the feasibility and acceptability of delivering educational video content as well as capturing feedback in drug users' natural environments. Dr. G. D. Kirk and colleagues report on implementation of ecological momentary analysis (EMA) methods using mobile devices among persons with a history of injecting drugs. During an intensive one-month study period, participants reported drug craving and use in realtime and displayed high levels of response to multiple daily EMA questionnaires regarding their mood, activity, and social and physical environment. Finally, Dr. A. Kurth and colleagues describe an mHealth intervention focused on PLWHA involved in the criminal justice system. In an ongoing randomized controlled trial, computer-delivered counseling is combined with text messaging to improve linkage, retention, and ART adherence during the critical transition following release from correctional settings. 

The populations under study in this special issue are highly diverse. Several studies focus on marginalized IDU populations from urban settings in the US. Two studies evaluate mHealth approaches among MSM who abuse methamphetamine. Another study examines technology-enhanced ART adherence support among IDUs in China. Implementation into these disparate populations emphasizes the broad reach of mHealth to cross socioeconomic, racial, or geographic boundaries. Further, successful demonstration of mHealth strategies in these diverse groups bodes well for implementation into other HIV and substance-using populations.

To summarize the findings reported in this special issue, despite the field being in early stages of development, mHealth holds substantial promise for optimizing HIV care and improving adherence to treatment. These strategies appear feasible and acceptable even among challenging, marginalized populations confronted by HIV and substance use. The next steps in development of mHealth intervention strategies will be challenging [8]. Substantial work is needed to develop the theoretical frameworks underpinning mHealth interventions. Further efforts will be required to refine real-time data collection and analysis procedures, identify the best methods for delivering context-sensitive interventions, maintain patient confidentiality, and determine the most appropriate methods for defining effectiveness in mHealth. Challenges exist regarding data security, staying current with rapidly evolving technologies, and best practices for interaction with market-driven industry partners. Despite these challenges, we remain strongly enthusiastic that increasingly available and affordable mHealth tools will continue to evolve and provide successful strategies to improve HIV care outcomes among PLWHA and substance abuse.

*Gregory D. Kirk* *Seth S. Himelhoch* *Ryan P. Westergaard* *Curt G. Beckwith*
